# Supplementary material for: Effects of different tennis racket string tension on forehand stroke effect and racket dynamic impact
Source: PLoS One. 2025 Jan 15;20(1):e0317442. doi: 10.1371/journal.pone.0317442 (PMC11734973; doi:10.1371/journal.pone.0317442)
Supplement: Attachment — Submitted filename: Review comment.pdf [file pone.0317442.s003.pdf]

Review comment

This manuscript entitled “Effects of different tennis racket string tension on forehand stroke effect and racket dynamic impact” is encouraging research, but there are several questions that should be addressed before this manuscript can be accepted for publication, which are listed below. You can revise this paper more properly.

Specific comments

1. I suggest optimizing the structure of this manuscript, the format and framework of this manuscript need to be optimized.
2. The format of the abstract part does not meet the specifications and it is recommended to modify it.
3. The citation format of the reference does not comply with the standards of academic articles. It is recommended to modify it.
4. The introduction section lacks the hypothesis of this study. It is recommended to state the problem of this study and add the research hypothesis.
5. The meaning and application of this research should be further and clearly expressed in the introduction section. It will help readers to understand the function and value of this study. Please further explain why have to do this research and the possible benefit of this research to this field.
6. Demographic information on subjects is missing, which is necessary material.
7. In the method section, comprehensive information about the experimental equipment needs to be added in the method section, such as brand, model, company, etc.
8. The title of the figure needs to be placed below the figure.
9. In the Methods section, please add more details and descriptions to complete this part.
10. The experiment is missing a lot of detailed descriptions and process descriptions, which violates the principle of repeatability of the experiment. It is recommended that the description of the method section be re-edited.
11. I suggest adding the key findings at the beginning of the discussion section.

12. The application of key findings in this study should be further mentioned and discussed.
13. The language level of this manuscript needs improvement, and I recommend that the manuscript undergo a thorough grammatical and formatting check.
