# Supplementary material for: Effects of different tennis racket string tension on forehand stroke effect and racket dynamic impact
Source: PLoS One. 2025 Jan 15;20(1):e0317442. doi: 10.1371/journal.pone.0317442 (PMC11734973; doi:10.1371/journal.pone.0317442)
Supplement: Attachment — Submitted filename: Review comment.pdf [file pone.0317442.s005.pdf]

Review comment

Thanks to the author for the revision of the manuscript. There are some comments as follows:

1. Line 40-42. In my point of view, this conclusion is unconvincing and not seriously. The 'stroke performance' in racket sports usually focuses on the stroke play motions and the performance of the athletes. However, this study only focuses on the impact of the racket on the ball's movement, so the use of "stroke performance" is not rigorous.

2. In the Line 98-99. '.... using a forehand stroke directed cross-court, Each participant…' I am confusing about this section, maybe you need change 'Each' to 'each'? Please check this issue of whole article.

3. In the Experimental Procedures section, the details are not enough to repeat this study. Please mentioned is there a ball machine or a coach to do the serve? If it is a machine or coach, how do you control the ball's condition? Such as ball's speed, fall, and rotation? Please, add more necessary details.

4. Line 116-127. Please check the format and font of this section. I noticed lots of font and formatting errors.

5. Line 167-170, There is a space in the line here, but there is no space between L184-L186, why?

6. Line 107. Please revise the citation format.

7. In the Table 2, 3, and 4, there are some error and formatting errors, please check it.

8. Please check the format and font of whole article again. Because I noticed there are lots of font formatting errors in the manuscript.
