# Supplementary material for: Effects of different tennis racket string tension on forehand stroke effect and racket dynamic impact
Source: PLoS One. 2025 Jan 15;20(1):e0317442. doi: 10.1371/journal.pone.0317442 (PMC11734973; doi:10.1371/journal.pone.0317442)
Supplement: Attachment — Submitted filename: Review comment.pdf [file pone.0317442.s008.pdf]

Review comment

Thank you for all the edits and adjustments you made to this manuscript. There are several questions that should be addressed which lists below. You can revise this paper more properly.

Specific comments

1. The manuscript mentions that the string tension of a tennis racket has an important influence on playing performance and material properties, but does not elaborate on why string tension is an important area of research. Some background information could be added, such as how string tension affects the performance of professional and amateur players and its importance in the selection of tennis equipment.
2. Please be more specific about why you chose forehand strokes as the subject of your study and why you chose the International Tennis Number (ITN) as the evaluation criterion.
3. L58-L59, 'Much of the prior research was conducted under laboratory conditions, lacking objective standards for evaluating stroke control based on participant performance' …The journal encourages the addition of articles with more recent years. The following literature is encouraged to be cited and referenced: 'He Y, Fekete G, Sun D, Baker JS, Shao S, Gu Y. Lower Limb Biomechanics during the Topspin Forehand in Table Tennis: A Systemic Review. *Bioengineering*. 2022; 9(8):336. https://doi.org/10.3390/bioengineering9080336'.
