# Supplementary material for: Effects of different tennis racket string tension on forehand stroke effect and racket dynamic impact
Source: PLoS One. 2025 Jan 15;20(1):e0317442. doi: 10.1371/journal.pone.0317442 (PMC11734973; doi:10.1371/journal.pone.0317442)
Supplement: Attachment — Submitted filename: Response to Reviewers.docx [file pone.0317442.s002.docx]

List of Responses

Manuscript Number: PONE-D-24-00255

Dear Editors and Reviewers:

Thank you for your letter and for reviewers’ comments concerning our manuscript entitled “Effects of different tennis racket string tension on forehand stroke effect and racket dynamic impact” (Manuscript Number: PONE-D-24-00255).Those comments are all valuable and very helpful for revising and improving our paper, as well as the important guiding significance to our researches. We have studied comments carefully and have made correction which we hope meet with approval. Revised portion are marked in red in the revised manuscript.

We have conducted comprehensive revisions to the original manuscript, and the responses to the reviewers’ comments are as flowing:

Responds to the reviewers’ comments:

**Reviewer #1:**

**General comments：**

**Comment 1:** the Author/s did a good job in writing a readable manuscript. This paper could be considered qualified to be published on Plos One if the authors apply the modifications requested, "Major issues" are recommended. It is the reason why "Major revision" is my personal choice. I would be happy to review a revised version of this manuscript.The aim of the submitted manuscript was to control explore the impact of changes in tennis racket string tension on hitting performance and racket dynamic response.This paper could be considered qualified to be published if the authors apply the modifications requested.

**Response 1: We appreciate the reviewer for the favorable analysis of our original submission and for highlighting the significance of our studies.**

**Comment 2:** The basic descriptive level adopted by author(s) does not seem compatible with interests of both researchers' conceptual challenges and coaches' practical applications to training. The main concern refers to the weak/absent theoretical background; there is no perceivable research question or rationale for the study presented to readers. Author(s) do not consider any specific difficulties or other aspects of performing these different types of tennis techniques to minimally justify the comparison; on the other hand, the use of the descriptive knowledge obtained from the observed differences was not discussed in terms of applications for athletes and coaches. In short, the manuscript is limited to a laboratory exercise to partially describe the participants' movement.

**Response 2: Thank you for your review comments. We have revised the introduction according to your suggestions. The first paragraph of the introduction now presents the research background, emphasizing the significance of string tension in impacting the effectiveness of the stroke and the dynamic response of the racket. The second and third paragraphs discuss the current research on the effects of string tension on the stroke outcome and the state of research on racket dynamics in terms of impact and vibration, as well as the shortcomings in these areas. The fourth paragraph summarizes these issues, noting the lack of quantitative control and evaluation standards in terms of stroke effectiveness, and the oversight of human involvement in practical situations in the research on impact and vibration.**

**In tennis, the forehand stroke is second only to the serve in importance, and forehand techniques can be divided into topspin and flat strokes. The flat stroke, being an important scoring method that requires high control precision, was the focus during our testing process. We asked all participants to use the forehand flat stroke technique to complete the tests, which eliminated the variability that different tennis techniques might introduce. specific difficulties and other aspects of performing these different types of tennis techniques have been discussion in line 300-309 of manuscript.**

**The article has been expanded to include discussions about the use of the descriptive knowledge obtained from the observed differences in terms of applications for athletes and coaches in line 285-296 of manuscript .**

**Specific comments:**

**Comment 1:** **Introduction. Line 71: the Aim of the study has to be included at the end of the Introduction section.**

**Response 1: The aim of the study has been included in the line 71-74 of manuscript.**

**Comment 2: Subject and Methods. Line 74: more detailed information about participants have to be included in this section (mean values and standard deviation): age, weight, eight,handedness, expertise (years), hours of training per week, etc.**

**Response 2: We are sorry for our unclear descriptions of the Participants , Participants detailed information are included in Table1 in the line 83 of manuscript**

**Comment 3:** **Line 86. A portable radar speed gun was employed. Details about it have to be reported in the manuscript: brand, model, etc.**

**Response 3: We are sorry for our unclear descriptions of the portable radar speed gun ,Details about the radar speed gun has been reported in the line 95-96 of manuscript**

**Comment 4: Figures 1 and 2 don’t reach a minimum standard of quality. The two pictures need to be removed from the paper and replaced by high quality pictures or figures/drawings.**

**Response4: According to the requirements of the journal picture, the quality of the picture has been modified. See the attachment for the modified picture.**

**Comment5: Line 83-85: there are many typing errors.**

**Response 5: We are sorry for typing errors. The typing errors has been corrected in lines85-87****of manuscript**

**Comment 6: Line 95: a feeding machine was used in the present investigation. The brand needs to be specified.**

**Response 6: We are sorry for our unclear descriptions of feeding machine ,In order to ensure that the subjects hit the ball with the maximum power of forehand, we used a self-made simple ball feeding device, and adjusted the device to make the ball in the most suitable hitting position, which can effectively avoid the instability of the machine feeding ball. The picture of the device is illustrated in the Fig3.**

**Comment 7:** **The balls were dropped by a feeding machine in a specific area of the tennis court. This area has to be described by Authors.**

**Response 7: We are sorry for our unclear descriptions of where is feeding machine, the specific area of the tennis court is described in the line98-101, which is shown by the triangle in Fig4**

**Comment 8:** **Line 97: information about target area (dimension and position) can not totally excluded from the paper. I would suggest to include a figure about the set-up.**

**Response 8: We are sorry for our unclear descriptions of target area, information about target area is shown in the Fig4**

**Comment 9:** **• Line 102: the subjects randomly used 48-54-60 lbs string tension. Authors have to justify this decision and support it by references.**

**Response 9: The racquet used for testing has a recommended racquet tension of 40-60 pounds, combined with previous studies（Fabre, 2014;Zhao, 2010; Bower, 2007;R.Cross, 2001）. So the 48-54-60 lbs string tension was selected**

**Comment 10: • Line 102. There are errors about unite of measure.**

**Response 10: Errors about unite has been corrected in lines 113 of	manuscript**

**Comment 11: Line 126: more details about “YSV data acquisition system” have to be included in the present section.**

**Response 11: The details about “YSV data acquisition system” included in the line 86-95 of manuscript**

**Comment 12:** **Line 139-139. The title of the sub-section needs to be modified. Indeed, “stroke effect” seems not to describe the date present in the Table 1.**

**Response 12: In the line 139-140, the title of the sub-section was modified to “Impact of different string tensions on the effectiveness of stroke”**

**Comment 13: Discussion: a Limits of the study section needs to be included in the present investigation.**

**Response 13: We apologize for the inappropriate discussion here, the Limits of the study was included in the line 301-310 of manuscript**

**Comment 14: Discussion: some practical applications have to be reported at the end of the manuscript**

**Response 14: We apologize for the inappropriate discussion here, the practical applications have been reported in line 288-300of manuscript**

**Comment 15: Line 261-262. This sentence has to be removed from the Conclusion and included in the Methods.**

**Response 15: Your suggestion is quite reasonable . The sentence in line 261-262 has moved from the Conclusion to line 101-102 in the Methods of manuscript**

**Reviewer #2: Thank you for the opportunity to review this unique study outlining tennis racket string tension. Overall, this manuscript is well-written, stating that the paper is interesting and correctly prepared.**

**Response 1: We appreciate the reviewer for the favorable analysis of our original submission and for highlighting the significance of our studies.**

**Specific comments:**

**Comment 1 for “Abstract”: In the abstract part, methods, it is essential to add the something about statistical analyses.**

**Response 1 for “Abstract”: We apologize for the inappropriate description here. statistical analyses was add to line20-21“All data were tested using paired samples T-test with a statistical significance level of P<0.05”**

**Comment 2 for “Abstract”: In the abstract part, results, main findings are needed to show and combine in this part.**

**Response 2 for “Abstract”: We apologize for the inappropriate description here. The results and mains findings have been showed and combine in the line 21-35**

**Comment 1 for “Introduction”: In the intro part, the first paragraph, “The tennis racket is a complex system composed of the frame, handle, and strings” It is recommended that add some relevant references here.**

**Response 1 for “Introduction”: “The tennis racket is a complex system composed of the frame, handle, and strings” was modified to “****The tennis racket is composed of the frame, handle, and strings” . The relevant references is Analysis and experimental research on the mechanical characteristics of tennis rackets**

**Comment 2for “Introduction”: In the intro part, display the aim of this study rather than the necessary and hypothesis**

**Response 2for “Introduction”: The aim of the study was displayed in the line 71-74 of manuscript**

**Comment for “Methold”:** **In the methold part, the method is well-structured, with a clear and correct development of sections. It is better to add a figure of experimental set-up which includes participant, racket, net and other necessary equipment.**

**Response for “Method”:The figure of experimental set-up was added in Fig1. Fig2. Fig3 , Also the description of the “Experimental equipment”section has also been modified, The details see the line 85-102 of manuscript**

**Comment for “Results”: In the results part, tables included in the paper are clear, and there is extensive notes accompanying them. This suggests that the visual representations are effective in conveying information. However, the content of the results text is too short, try to add some details in order this section entirely.**

**Response for “Results”:The details have been added in the line 144-158,165-176,183-189**

**Comment for “Discussion”: In the discussion part, the authors should display the limitations of their study, which is appropriately discussed in the end.**

**Response for “Discussion”:** **We apologize for the inappropriate description here. The Limits of the study have been displayed in line 301-310, and discussed appropriately**
