# Supplementary material for: Effects of different tennis racket string tension on forehand stroke effect and racket dynamic impact
Source: PLoS One. 2025 Jan 15;20(1):e0317442. doi: 10.1371/journal.pone.0317442 (PMC11734973; doi:10.1371/journal.pone.0317442)
Supplement: Attachment — Submitted filename: Response to Reviewers.docx [file pone.0317442.s004.docx]

**List of Responses**

Manuscript Number: PONE-D-24-00255R1

Dear Editors and Reviewers:

Thank you for your letter and for reviewers’ comments concerning our manuscript entitled “Effects of different tennis racket string tension on forehand stroke effect and racket dynamic impact” (Manuscript Number: PONE-D-24-00255R1).Those comments are all valuable and very helpful for revising and improving our paper, as well as the important guiding significance to our researches. We have studied comments carefully and have made correction which we hope meet with approval. Revised portion are marked in red in the revised manuscript.

We have conducted comprehensive revisions to the original manuscript, and the responses to the reviewers’ comments are as flowing:

Responds to the reviewers’ comments:

**Reviewer #2:** he revised manuscript is well-written and it is able to be considered to publish. However, some issues have to be modified before publication.

**Specific comments:**

**Comment 1: The format of cited references in the introduction, such as Zhang W, Jean Bernard Fabre and others, needs to be revised.**

**Response 1: The format of cited references in the introduction has been revised in the line 59-74**

**Comment 2: Do not use "meanwhile" in the research article, it is a colloquial expression**

**Response 2: I’m sorry to this error, the mistake has been revised.**

**Comment 3: If humans were involved in the study, it is recommended use "participant" not "subject" in the method.**

**Response 3: Your profound language skills are worthy of my learning, and I have made modifications in the line 90**

**Comment 4: All the figures are not only blurry but chaotic background. It is better to change or replace figures.**

**Response4: According to your suggestion, we have corrected the figures**

**Comment5: It is feasible to combine the "Experimental equipment" with "Testing method" which could be called "Experimental Procedures".**

**Response 5: Thank you for your valuable advice, the "Experimental equipment" with "Testing method" have been calles "Experimental Procedures" in line 102-135**

**Comment 6: The writing is of poor quality, please proof-read to improve the clarity for reading and understanding.**

**Response 6: I’m sorry for that, we have improved the writing quality**

**Reviewer #3: This manuscript entitled “Effects of different tennis racket string tension on forehand stroke effect and racket dynamic impact” is encouraging research, but there are several questions that should be addressed before this manuscript can be accepted for publication, which are listed below. You can revise this paper more properly.**

**Specific comments:**

**Comment 1: I suggest optimizing the structure of this manuscript, the format and framework of this manuscript need to be optimized.**

**Response 1: Thank you for your valuable advice, we have optimizing the structure of this manuscript in the "Abstract","Methods","** **Discussion"," References"**

**Comment 2: The format of the abstract part does not meet the specifications and it is recommended to modify it.**

**Response 2: I’m sorry for the mistake, the format of the abstract part has been modified in line 37-47, also check the format of the manuscript.**

**Comment 3: The citation format of the reference does not comply with the standards of academic articles. It is recommended to modify it.**

**Response3: PLOS uses the reference style outlined by the International Committee of Medical Journal Editors (ICMJE), also referred to as the “Vancouver” style, according to the “Vancouver” style, we modified the citation format of the reference in the line 344-448**

**Comment 4: The introduction section lacks the hypothesis of this study. It is recommended to state the problem of this study and add the research hypothesis.**

**Response 4: Thank you for you advise, we added the research hypothesis in the line84-88**

**Comment 5: The meaning and application of this research should be further and clearly expressed in the introduction section. It will help readers to understand the function and value of this study. Please further explain why have to do this research and the possible benefit of this research to this field.**

**Response 5: Thank you for your comment, we added the meaning and application of this research in line 85-88, we have explained why have to do this research in line 56-79**

**Comment 6: Demographic information on subjects is missing, which is necessary material.**

**Response 6:We have already added demographic information in line 91-101**

**Comment 7: In the method section, comprehensive information about the experimental equipment needs to be added in the method section, such as brand, model, company, etc.**

**Response 7: Than you for you comment, we added the comprehensive about the experimental equipment in line 117-128**

**Comment 8: The title of the figure needs to be placed below the figure.**

**Response 8: I’m sorry for that, PLOS Manuscript Body Formatting Guidelines in line 32-34 demand that “Each figure caption should appear directly after the paragraph in which they are first cited.” “Figures should be uploaded separately as individual files.” So the figure title and caption can appear in the Manuscript.**

**Comment 9: In the Methods section, please add more details and descriptions to complete this part.**

**Response 9: You comment is very important, we have written the Methods in line 91-134**

**Comment 10: The experiment is missing a lot of detailed descriptions and process descriptions, which violates the principle of repeatability of the experiment. It is recommended that the description of the method section be re-edited.**

**Response 10: Thank you for your advice, the detailed descriptions and process descriptions have been re-edited in line 103-152**

**Comment 11: I suggest adding the key findings at the beginning of the discussion section.**

**Response 11: Thank you for your valuable advice, the key findings has been added at the beginning of the discussion section in line 205-211**

**Comment 12: The application of key findings in this study should be further mentioned and discussed.**

**Response 12: The application of key findings in this study should be further mentioned and discussed in line 307-321**

**Comment 13: The language level of this manuscript needs improvement, and I recommend that the manuscript undergo a thorough grammatical and formatting check.**

**Response 13: I’m sorry for may poor language of this manuscript, In accordance with your suggestions and the professional polishing team, we have made changes to the grammar and format of the manuscript**
