# Supplementary material for: Effects of different tennis racket string tension on forehand stroke effect and racket dynamic impact
Source: PLoS One. 2025 Jan 15;20(1):e0317442. doi: 10.1371/journal.pone.0317442 (PMC11734973; doi:10.1371/journal.pone.0317442)
Supplement: Attachment — Submitted filename: Response to Reviewers.docx [file pone.0317442.s006.docx]

**List of Responses**

Manuscript Number: PONE-D-24-00255R3

Dear Editors and Reviewers:

Thank you for your letter and for reviewers’ comments concerning our manuscript entitled “Effects of different tennis racket string tension on forehand stroke effect and racket dynamic impact” (Manuscript Number: PONE-D-24-00255R1). Those comments are all valuable and very helpful for revising and improving our paper, as well as the important guiding significance to our researches. We have studied comments carefully and have made correction which we hope meet with approval. Revised portion are marked in red in the revised manuscript.

We have conducted comprehensive revisions to the original manuscript, and the responses to the reviewers’ comments are as flowing:

Responds to the reviewers’ comments:

**Reviewer #2:** • **The introduction effectively presents the motivation and provides basic information, referring to other publications in the field.**

**• The publication is well-structured, with a clear and correct development of sections, including abstract, introduction, materials and methods, results, discussion, and conclusions.**

**• The graphs included in the paper are clear. This suggests that the visual representations are effective in conveying information.**

**• The authors demonstrate awareness of the limitations of their study, and this is appropriately discussed in the discussion section.**

**• The overall conclusion is positive, stating that the paper is interesting and correctly prepared. The only suggested improvement is related to the volume of the abstract, which is too long.**

**• After decreasing the volume of abstract, the paper is suitable for publication.**

**Response: Thank you for your comments, we decreased the volume of abstract in line 14-39**

**Reviewer #3: Thanks to the author for the revision of the manuscript. There are some comments as follows:**

**Specific comments:**

**Comment 1: Line 40-42. In my point of view, this conclusion is unconvincing and not seriously. The ‘stroke performance’ in racket sports usually focuses on the stroke play motions and the performance of the athletes. However, this study only focuses on the impact of the racket on the ball’s movement, so the use of "stroke performance" is not rigorous.**

**Response 1: Thank you for your advice, according your view, “stroke performance” was change to “stroke effect” in line 31-32.**

**Comment 2: In the Line 98-99. ‘.... using a forehand stroke directed cross-court, Each participant…’ I am confusing about this section, maybe you need change ‘Each’ to‘each’? Please check this issue of whole article.**

**Response 2: I’m sorry for the mistake, we modified this in line 88-89, and check the issue of whole article.**

**Comment 3: In the Experimental Procedures section, the details are not enough to repeat this study. Please mentioned is there a ball machine or a coach to do the serve? If it is a machine or coach, how do you control the ball’s condition? Such as ball’s speed, fall, and rotation? Please, add more necessary details.**

**Response3: Before the test, we** **Before the formal test, we conducted a pre-experiment, adjusted the self-made ball feeding device to ensure the quality of the ball. The self-made ball feeding device is shown in the figure 2, the details description is shown in line 111-117**

**Comment 4: Line 116-127. Please check the format and font of this section. I noticed lots of font and formatting errors.**

**Response 4: Thank you for your comment, we revised the format and font of this section in the line104-119. The figure captions in line 111,114,118 use bold type for the figure titles according the manuscript body formatting guidelines.**

**Comment 5: Line 167-170, There is a space in the line here, but there is no space between L184-L186, why?**

**Response 5: I’m sorry for the stupid mistake, we correct the mistake and check the hole manuscript**

**Comment 6: Line 107. Please revise the citation format.**

**Response 6: Thank you, We have already revised the citation format in line 95-96**

**Comment 7: In the Table 2, 3, and 4, there are some error and formatting errors, please check it.**

**Response 7: Thank you for the comment, we revised the error in the Table 2,3,4.**

**Comment 8: Please check the format and font of whole article again. Because I noticed there are lots of font formatting errors in the manuscript.**

**Response 8: Thank you for this comment, We check the full manuscript and modified the errors.**
