# Supplementary material for: Effects of different tennis racket string tension on forehand stroke effect and racket dynamic impact
Source: PLoS One. 2025 Jan 15;20(1):e0317442. doi: 10.1371/journal.pone.0317442 (PMC11734973; doi:10.1371/journal.pone.0317442)
Supplement: Attachment — Submitted filename: Response to Reviewers.docx [file pone.0317442.s007.docx]

**List of Responses**

Manuscript Number: PONE-D-24-00255R3

Dear Editors and Reviewers:

Thank you for your letter and for reviewers’ comments concerning our manuscript entitled “Effects of different tennis racket string tension on forehand stroke effect and racket dynamic impact” (Manuscript Number: PONE-D-24-00255R3). Those comments are all valuable and very helpful for revising and improving our paper, as well as the important guiding significance to our researches. We have studied comments carefully and have made correction which we hope meet with approval. Revised portion are marked in red in the revised manuscript.

We have conducted comprehensive revisions to the original manuscript, and the responses to the reviewers’ comments are as flowing:

**Responds to the reviewers’ comments:**

**Comment 1:**

Is the manuscript technically sound, and do the data support the conclusions? The manuscript must describe a technically sound piece of scientific research with data that supports the conclusions. Experiments must have been conducted rigorously, with appropriate controls, replication, and sample sizes. must be drawn appropriately based on the data presented.

**Reviewer #3:** Partly

**Response：**We are sorry for our mistakes. After examining our manuscript, we found there may be some unclear and insufficient descriptions of the results and conclusions, especially in the Abstract section.

Therefore, we enriched our results, and rewrote the conclusions to make them correspond to our results in lines 35-42.

**Comment 2:**

**Reviewer #3:** There are still some issues about format should be revised. Please check the whole manuscript again, thanks.

**Response：**Sorry for the issues about format, and we have made some modifications in the manuscript, such as below:

1) In line 114: the brand of tennis balls used in the test was described more precision and clarity.

2) In Table 4 and lines 190-191: we used more standardized and accurate statistical significance annotation symbols.

3) In lines 322-328: we rewrote the conclusions to make them correspond to our results.
